# Electrochemical Deposition of Ni, NiCo Alloy and NiCo–Ceramic Composite Coatings—A Critical Review

**DOI:** 10.3390/ma13163475

**Published:** 2020-08-06

**Authors:** Nyambura Samuel Mbugua, Min Kang, Yin Zhang, Ndumia Joseph Ndiithi, Gbenontin V. Bertrand, Liang Yao

**Affiliations:** College of Engineering, Nanjing Agricultural University, Nanjing 210031, China; 2017112123@njau.edu.cn (N.S.M.); 2018212003@njau.edu.cn (Y.Z.); 2017112122@njau.edu.cn (N.J.N.); 2019212016@njau.edu.cn (G.V.B.); 2018112003@njau.edu.cn (L.Y.)

**Keywords:** nanostructured coatings, microhardness, corrosion resistance, electrochemical deposition

## Abstract

In recent years, alloy and alloy-ceramic coatings have gained a considerable attention owing to their favorable physicochemical and technological properties. In this review, we investigate Ni, NiCo alloy and NiCo–ceramic composite coatings prepared by electrodeposition. Electrodeposition is a versatile tool and cost-effective electrochemical method used to produce high quality metal coatings. Surface finish and tribological properties of the coatings can be further improved by the addition of suitable agents and control of deposition operating conditions. In this review, Ni, NiCo alloy and NiCo–ceramic composite coatings prepared by electrodeposition are reviewed by critically evaluating previous researches. The use of the coatings and their potential for future research and development are discussed.

## 1. Introduction

Materials are a fundamental pillar in engineering technology. The materials’ electrochemical, thermal and mechanical interaction begins on the surface. However, material surfaces face the constant threat of wear and corrosion resulting in massive losses in industry. The use of surface enhancement technology to prevent or mitigate the loss has therefore become inevitable. Over the last few decades, major scientific development in the fields of metallurgy and materials has occurred, giving rise to new engineering materials with superior properties. Developing suitable processing to produce desired materials is complex and requires altering the inherent properties of the materials. Consideration is made of the overall economic perspective and its environmental impact.

Electrodeposition is an electrochemical process that is used to modify the surface structure. Use of electrodeposition in surface engineering can be traced to nearly 200 years ago based on some hypotheses [1]. The galvanic cell, invented in early 1800′s, paved way for use of electric current to produce coatings as a more cost-effective technique. The electrodeposition technique possesses an edge over other coating techniques due to several reasons [2]:

(i) Low initial investment coupled with high rate of production.
(ii) It can be used with a wide variety of shapes and sizes of substrates
(iii) Ease of producing economically viable quantities of nanocomposite materials, with grain sizes as small as 10 nm.
(iv) Products of electrodeposition require no further processing and can be used immediately after the process.
(v) It is an easy concept that can be replicated in industry and laboratories with minimal technological barriers.
(vi) Electrodeposited Ni coatings have exhibited superior density and lower porosity.

Electrodeposition is based on the principle that a layer of coating, either single or multilayer, forms as a result of the electrode–electrolyte electrochemical reactions occurring leading to electrodeposition of ions contained in electrolyte. It utilizes the properties of materials in their metastable state as a result of reduction of the grain size on the nano scale. In such a state, the grain boundaries contain a proportion of atoms that is higher or equivalent to those inside the grains [3]. These new types of materials consisting of nanoparticles are referred to as nanocomposite materials and they exhibit superior properties compared to traditional grain sized materials and sometimes offer completely new properties altogether. As a result, nanocomposite materials with grain sizes below 100 nm have received considerable attention from scientists and researchers all over the world. Several new electrodeposition synthesis methods have been devised over the years to increase production efficiency of new materials and minimize cost. This has ranged from using the basic direct current set ups to more ambitious procedures such as: pulse plating, jet electrodeposition, and the pulse reversal current technique [4,5,6].

Pure Ni is one of the most widely used alloying metals in the world owing to its superior corrosion and wear resistance [7]. Electrodeposited Ni coatings have uses in decoration, functional uses, as well as in engineering for surface protection [8]. Superplasticity in metals (materials exhibiting increased elongations to failure of >500%) is dependent on elevated testing temperatures and fine grain sizes of <10 μm [9]. Prasad and Chokshi [9] reported that electrodeposited nanocrystalline Ni is characterized by good superplasticity properties and is used in studying the phenomenon in electrodeposited metals owing to the ease of synthesizing coatings with small grain sizes. Ni coatings exhibit improved resistance to localized corrosion and this makes them perfect for use as anti-corrosive coatings [2]. Furthermore, research shows that thin films of different materials coated with epitaxial thin Ni coatings of a few nanometers have similar hardness to bulk nickel, such that the wear resistance of micro/nano electro mechanical system devices can be greatly improved if coated with a thin Ni coating [10]. Studies have shown that nano-sized nickel can aid the diamond yielding process. Ni atoms have a three dimension absent state which serves to attract electrons in the carbon fullerenes. This in turn causes the sp2 fullerene to be transformed into a diamondlike sp3 structure. This transformation can also be attributed to high reactivity which effectively aids the change at an impulse during shock wave loading [2].

Ni based alloy coatings can also be produced using electrodeposition. Choice of the alloying metal depends on the properties desired, which can range from good electrical conductivity, good wear and corrosion resistance, soft magnetic properties to special optical properties. Over the years, many different types of metals have been electrodeposited with Ni to form alloys: Co, Fe, Cu and W.

Ni–Co is one such alloy. There exist several different synthesis techniques for Ni–Co alloy coatings and they include: radio frequency magnetron sputtering [11], electrodeposition [12] and vacuum evaporation [13,14]. The electrodeposition technique has several advantages over the other two methods and they include: low cost, simplicity, scalability and manufacturability [15]. Furthermore, electrodeposition can be used to grow a wider range of materials.

Research shows that electrodeposited Ni–Co alloy coatings exhibit better properties compared to pure Ni coatings [12]. Ni–Co alloy exhibits higher hardness, better adhesion, excellent magnetic properties, high wear and corrosion resistance as well as good stability at high temperature [16,17,18,19]. Wang et al. [20] researched the effect of cobalt content on mechanical and microstructural properties on Ni–Co alloy coatings. It was reported that Ni–Co alloy coatings exhibited approximately double the microhardness when compared to pure Ni coatings. It was also reported that the Ni–49Co coatings exhibited a decreased rate of wear in comparison to pure Ni coatings. Hassani et al. [21] researched on low temperature superplasticity of nanocrystalline electrodeposited Ni–Co alloy with an average grain size of 20 nm. It was reported that a maximum elongation of 279% at a temperature of 773 K was obtained. Ni–Co alloys are considered to be the best suited materials for replacing hard chromium [22]. Research shows that microhardness in Ni–Co alloys increases gradually with increase in Co content up to an optimum level, after which the microhardness decreases with further increase in Co content [16].

To further improve the properties of Ni–Co alloy coatings, nanoparticles have been suspended in electrolyte and they become embedded into the electro-formed solid phase layer during electrodeposition [23]. In such materials, the inherent properties of the nanoparticles have been found to significantly influence the overall properties of the nanocomposite coatings. Many different types of nanoparticles have been electrodeposited with Ni–Co alloy including SiC, Al_2_O_3_, SiO_4_, ZrO_2_, Cr_2_O_3_, Si_3_N_4_ and TiO_2_ [24]. These nano particles used in electrodeposition can be classified as either hard materials or soft materials depending on the desired properties. Soft materials such as graphite offer properties which include lower the friction coefficient to reduce wear and the coefficient of friction between shearing surfaces [25]. Hard materials such as Al_2_O_3_ improve the microhardness and wear resistance of surfaces [26]. The matrix phase microstructure coupled with nanoparticle content and distribution in the metallic matrix phase significantly influences the properties of nanoparticle reinforced Ni–Co matrix nanocomposite coatings. From past research, it is clear that addition of nanoparticles has served to improve the properties of Ni–Co coatings. In some instances, however, addition of Co has been observed to improve the overall properties of Ni-nanocomposite coatings such as hardness and residual stress [27]. Addition of Co^2+^ in Ni/diamond plating baths has been seen to greatly improve the deposition of diamond in the coatings resulting in higher diamond content, enhance bonding between matrix and particles, as well as more uniformly dispersed particles in the metal matrix, resulting in improved wear resistance and hardness properties [28]. The properties of Ni–Co alloys and their composite coatings have also been previously reported on by Karimzadeh A et al. [29]. This review aims takes a different approach to the wide research field of Ni–Co and Ni–Co-based composite coatings. Factors such as the effect of electrode orientation and forces existing in the electrolyte bath on the deposition process have been considered. Moreover, additives such as boric acid have been extensively explored, and the intricate working of the Watts solution has been more deeply discussed for easier understanding. Properties such as adhesion between substrate and coating have been extensively discussed, comparisons between techniques drawn, and recommendations for further adhesion improvement have been presented.

## 2. Electrodeposition Methods

### 2.1. Direct Current Electrodeposition

In Direct current (DC) electrodeposition, an electric current is continuously transferred through the system without any interruptions. DC electrodeposition is divided into two types depending on the orientation of the electrodes in electrolyte during the electrodeposition process. These are the conventional electrodeposition, and sediment codeposition (SCD) techniques. For conventional electrodeposition, the electrodes are placed vertically in the electrolyte, but for SCD they are placed horizontally. Adsorption of nanoparticles into the alloy matrix during electrodeposition is greatly influenced by forces acting on the suspended nanoparticles. The kinetics involved can be used to explain this difference. The two main forces at work during SCD electrodeposition are gravitational pull and the electrophoresis force, thereby giving more desirable properties compared to conventional deposition which solely relies on gravitational pull [30]. DC has several advantages over pulse electrodeposition and pulse reversal current (PRC) electrodeposition. These include simplicity, the availability of vast technical knowledge and affordability. Borkar T [31] reported that for all Ni and Ni nanocomposite coatings deposited, DC deposited coatings exhibited much stronger (less random) crystallographic textures compared to coatings deposited using PC and PRC deposition techniques. Furthermore, in DC electrodeposition, the Co content in the deposited Ni–Co coatings is dependent on the composition of the electrolyte, unlike in jet electrodeposition where Co electrodeposition is controlled by diffusion [4].

### 2.2. Pulse Current Electrodeposition

Pulse current electrodeposition (PC) has been used extensively over the years in Ni–Co electrodeposition [32]. The three most fundamental parameters that affect the properties of the coatings include in pulse current electrodeposition include: peak current ($I_{peak}$), pulse imposition time (ON-time, $T_{ON}$) and switch off time (OFF-time, $T_{OFF}$). These parameters relate mathematically to evaluate (1) pulse frequency, (2) duty cycle, and (3) average current density, as shown [32,33]:

(1) $$f=\frac{1}{T_{OFF}+T_{ON}}$$

(2) $$\gamma =\frac{T_{ON}}{T_{OFF}+T_{ON}}*100$$

(3) $$I_{avg}\;\frac{T_{ON}}{T_{OFF}+T_{ON}}*I_{peak}=I_{peak}$$

where γ is the duty cycle, f the frequency, $I_{peak}$ the peak current density, and $I_{\mathrm{avg}}$ the average current density.

In pulse electrodeposition, peak current density has a significant effect on microhardness, crystallite size, surface morphology, microstructure, composition, and tensile strength of PC deposited Ni–Co alloys and their nanocomposites [34]. Crystallization and growth in turn determine the microstructure of the nanocomposite. The texture of the coatings is determined by both peak current density and organic surfactants [35]. The quantity of adatoms located at the surface is higher due to the applied higher current density as compared to DC electrodeposition. This results in smaller grain sizes [5]. Padmanabhan [36] reported that pulse electrodeposition is used as an effective method for the reduction of grain sizes to nanoscale [5].

Although it is a relatively low-cost synthesis technique with simple implementation, pulse electrodeposition is ideal for production of full density nanocrystalline materials [33]. Yang and Cheng [32] reported that the morphology of the deposited coatings changed from nodular to acicular, and a finer grain size was observed with increasing pulse frequency and decreasing duty cycle. It has also been reported that at low current densities, smoother surface morphologies are observed [37], but an increase in peak current density produces distinct colony-like morphologies characterized by clearer colony boundaries [34].

Pulse electrodeposition exhibits several advantages over DC electrodeposition, such as improved wear resistance and hardness, particle distribution, structure, morphological structure and the ability to control the grain sizes of the deposits [38]. PC electrodeposition has higher instantaneous current density compared to DC electrodeposition, thereby increasing its effectiveness in agitating the adsorption–desorption processes that occur at the Ni electrolyte interface. This makes it possible to control the electrodeposited Ni coating microstructure [39,40]. It has also been reported that PC deposits contain a higher nanoparticle content compared to coatings deposited by the DC deposition technique [31]. Yang and Cheng [32] concluded that the high microhardness of deposited Ni–Co–SiC coatings was primarily associated with increasing SiC content in the coatings with increasing pulse frequency and decreasing duty cycle. This significantly increased the microhardness as a function of the dispersion strengthening mechanism. Watts type baths used for pulse plating of Ni based coatings have been reported to produce the best coating results [2].

The PC deposition technique suffers from a drawback called the double layer capacitance effect. The double charging layer of the electrodes is subjected to charging and discharging occurrences. In a case where the ON- and OFF-times are much shorter than the charging and discharging times, the PC would revert to DC current. As such, care is taken to select a high frequency thus ensuring the effect of the double layer capacitance effect is negligible.

### 2.3. Jet Electrodeposition

**Figure 1:**
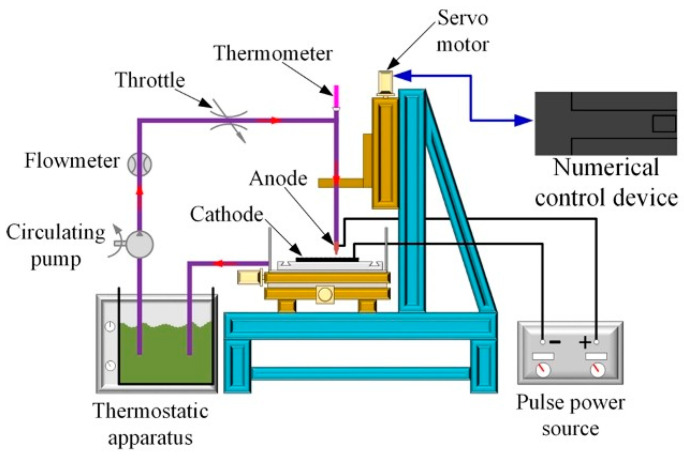
Schematic image of pulse jet electrodeposition.

In this technique, a jet of plating solution is directed at the cathode surface directly. There exists an electrical field between the anode (located in the nozzle) and cathode (substrate). Figure 1 is a schematic representation of jet electrodeposition technique setup [4].

As the plating solution flows, electric current is transferred along the stream of fluid to the substrate surface, thereby enabling deposition to occur on the cathode surface where the jet flows over [41]. Jet electrodeposition is a high-speed electroplating technique that offers a wide range of advantages [4]. These include: (i) a higher deposition rate compared to other conventional electrodeposition techniques, and (ii) a more efficient grain size refining effect. This is attributed to the cathode in the jet technique having a larger overpotential that can be used simultaneously with higher current densities. In this technique, Co content in the coatings increases with increases in electrolyte jet speed, Co^2+^ ion concentration in the electrolyte, and cathodic current density.

### 2.4. Pulse Reversal Current Electrodeposition

There are nine waveform parameters that can be used to represent the waveform characteristics according to the definition of bidirectional pulse parameters: (i) forward conduction time $t_{c}$, (ii) reverse conduction time $t_{d}$, (iii) forward peak current density $I_{p+}$, (iv) reverse peak current density $I_{p-}$, (v) average current density $I_{av}$, (vi) pulse period T, (vii) reverse current coefficient x, (viii) duty cycle *λ*, and (ix) frequency f [42]. The nine parameters are interrelated and they do not change independently. Where average current density $I_{av}$, duty cycle λ, reverse pulse coefficient x, and frequency f are considered as the independent variables, the parameters can be related mathematically, as shown in Equations (4)–(12) [43]:

(4) $$I_{p+}=\frac{I_{av}}{x\lambda +\lambda -x}$$

(5) $$I_{p-}=xI_{p+}$$

(6) $$t_{c}=\lambda T=\frac{\lambda }{f}$$

(7) $$t_{d}=T-t_{c}=\frac{1-\lambda }{f}$$

(8) $$T=t_{c}+t_{d}$$

(9) $$I_{av}=\frac{t_{c}\;I_{p+}-t_{c}\;I_{p-}}{T}$$

(10) $$\lambda =\frac{t_{c}}{t_{d}}$$

(11) $$x=\frac{I_{p-}}{I_{p+}}$$

(12) $$f=\frac{1}{T}$$

**Figure 2:**
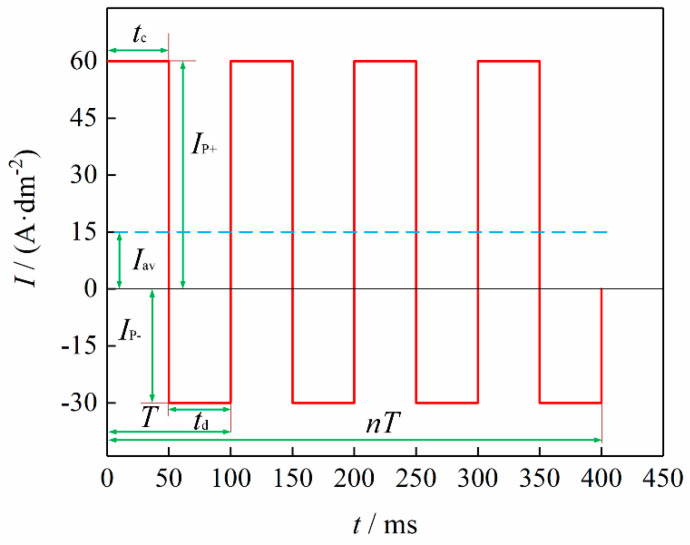
Schematic diagram of pulse waveform when $I_{av}$ = 15 A·dm^2^, λ = 0.5, x = 0.5, and f = 10 Hz.

Figure 2 shows the schematic diagram of a typical pulse-current wave form when $I_{av}$ = 15 A·dm^2^, λ = 0.5, x = 0.5, and f = 10 Hz.

Pulse reversal current (PRC) electrodeposition has been used over the years owing to its unique ability to enhance nanoparticle incorporation in electrodeposited composite coatings, with the highest reported content being 23 wt% [44,45,46]. Ni–Co coatings produced using the PRC synthesis technique have been reported to exhibit higher hardness, better anti-wear properties, more compact surfaces, lower residual macro stress and better compact surfaces than those produced using DC and PC electrodeposition techniques [6,40]. In particular, the hardness improvement has been attributed to increased distribution of nanoparticles in the deposited Ni–Co matrix. As for lower residual macro stress, PRC deposits are characterized by uniform coatings since the technique hinders thickening of the corners and edges, as is common with DC electrodeposition. It can also be postulated that adsorption of H atoms by the deposited coatings is suppressed by pulse intervals and reversal current in PRC technique [40]. PRC deposited Ni–Co nanocomposite coatings possess smoother surfaces, finer Ni matrix crystals, and smaller grain sizes compared to DC technique deposited coatings [40].

The smooth surfaces coupled with high hardness increase the load-carrying capacity of Ni–Co nanocomposite coatings and thereby improves their wear resistance properties. The lower macro-residual stress also plays a great part in wear resistance, where it causes lower brittleness and higher toughness in the coatings, and this decreases the rate of nanoparticle flaking and metal matrix peeling during wear testing [40]. PRC technique can be used in ultrasonic power conditions and this presents desirable effects on deposited Ni–Co coatings [47,48]. Overall, it presents several advantageous effects on the electrochemical process:

(i) Hindering sundries adsorption thereby altering the reaction mechanism,
(ii) An increase in exchange current density,
(iii) Lowering cathodic polarization,
(iv) Current efficiency and yield improvements, and
(v) (Strengthening conversion and diffusion.

It has been reported that Ni–Co nanocomposite coatings electrodeposited using the PRC technique subject to ultrasonic conditions present finer grained, compact, and uniform coatings [6].

## 3. Electrodeposition Parameters for Ni–Co Alloys

### 3.1. Effect of Co Concentration in Electrolyte

Cobalt content has been reported to increase with increase in Co concentration in the electrolyte bath [12,49]. For Ni–Co deposition, Ni and Co combine to form a solid solution that engulfs the nanoparticles suspended in the electrolyte. As such, the higher the concentration of Co element in the electrolyte, the more formation of this solid solution matrix occurs on the cathode surface. Variation of Co^2+^ in the electrolyte has significant effects on grain sizes, and cobalt content of the electrodeposited Ni–Co alloys and nanocomposites. Grain refinement can be explained using two approaches. Firstly, a lattice strain is produced as a result of the difference in atomic sizes of Co and Ni. As the content of Co increases, the lattice distortion becomes aggravated causing vacancy and dislocation defects in the lattice. Grain size refinement is a direct consequence of these defects.

Secondly, as a result of anomalous deposition of Co, the deposited Co content is higher than the corresponding concentration of Co in the electrolyte [49,50]. In this process, the less noble element (Co in this case) is reduced preferentially, resulting in a much higher content. This increase in content reaches a critical value, beyond which the microstructure of the electrodeposited coating changes from single phase (a-phase) face-centered cubic (FCC) to a combination of a-phase and hexagonal close-packed (HCP) e-phase. This combination of two structural phases results in grain refinement of deposited coatings.

Addition of Co^2+^ into the electrolyte also enhances transport and deposition of suspended nanoparticles. Transfer rate of nanoparticles through the bulk of the electrolyte is a function of electrophoretic forces existing between charged nanoparticles and the cathode surface which in turn is influenced by the quantity of adsorbed cations on the nanoparticle surfaces (zeta potential). Therefore, the amount and type of adsorbed cations determines the magnitude of electrophoretic force [51]. It was reported that deposition of nanoparticles was enhanced by addition of Co^2+^ into the electrolyte, and it can be suggested that zeta potential in Co^2+^ containing baths is much more positive, owing to Co^2+^ being much more easily adsorbed onto the surfaces of nanoparticles than Ni^2+^. As a result, the electrophoretic forces exerted on nanoparticles are more intense.

### 3.2. Current Density

Current density (CE) affects the current efficiency of the process. Current efficiency (%) describes the ratio of electrochemical current density for a specific reaction to total applied current density. It illustrates the transfer efficiency of electrons to the electrochemical system. Current efficiency (η) is calculated by factoring charge passed, weight of deposits given by the difference between the weight of samples before and after deposition, and the chemical composition of the coatings as shown in Equation (13) [52].

(13) $$\eta =\frac{W}{It}\left(\sum \frac{\left(Fg_{i}e_{i}\right)}{KN_{i}}\right)\times 100$$

where *W* is the weight of the deposit (g), *I* is the current passed (A), *t* is the deposition time (h), $g_{i}$ is the weight fraction of the element in the binary alloy deposit, $e_{i}$ is the number of electrons transferred in the reduction of 1 mol atoms of that element, $N_{i}$ is the atomic weight of that element (g/mol), *F* is the Faraday constant (96,485.3 C/mol) and *K* is a unit conversion factor (3600 C/A h).

Grain size, microstructure, brightness, thickness distribution, composition, surface morphology, microhardness and tensile strength of PC electrodeposited Ni–Co alloys are significantly influenced by variation of peak current density $J_{p}$ [34,53]. It has been reported that current density has a significant influence on rate of deposition, plating adherence and the quality of plating of Ni–Co coatings. The deposition rate increases with increases in current density [54]. Li et al. [34] used a pulse technique to research the effect of varying pulse frequency in electrodeposition of nanocrystalline Ni–Co deposits. It was found that increase in peak current density resulted in a lower cobalt content, smaller grain size, higher tensile strength and a colony like morphology. A recommendation for a peak current density range of 100–200 A·dm^−2^ was suggested for grain sizes ranging from 15–20 nm, a 7%–8% cobalt content, 590–600 kg mm^−2^ microhardness and 118–1200 MPa tensile strength. When using the PC technique to electrodeposit Ni–Co, it was reported that the lowest current densities achieved the most compact layer of alloys [5].

**Figure 3:**
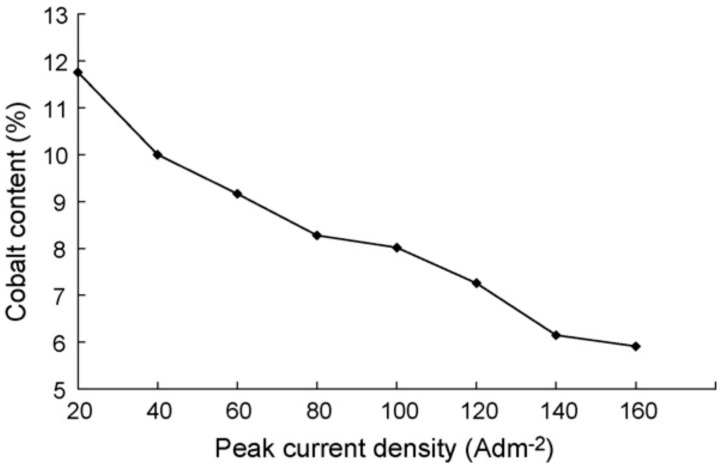
Cobalt content in Ni–Co alloy coatings with varying peak current density [34]. Reprinted from Applied Surface Science, 254, Yundong Li, Hui Jiang, Weihua Huang, Hui Tian, Effects of peak current density on the mechanical properties of nanocrystalline Ni–Co alloys produced by pulse electrodeposition/Pages No. 6865–6869, Copyright (2020), with permission from Elsevier.

Extremely high current density $J_{p}$ however has been reported to have a drastic effect on microhardness and tensile strength of electrodeposited Ni–Co coatings [34]. This has also held true for Ni–Co alloys electrodeposited using jet electrodeposition technique [4]. It could be suggested that this is because of the decrease in cobalt content with increase in the current density. Li et al. [34] researched the effects of peak current density on mechanical properties of Ni–Co alloys and reported that Co content decreased with peak current density increase. One of the key aspects in electrodeposited Ni–Co alloys and nanocomposite coatings is solid solution strengthening where the Ni and Co in electrolyte combine to form a solid solution. A decrease in Co content will therefore yield less solid solution strengthening for coatings electrodeposited at higher current densities. Figure 3 shows the relationship between peak current density and cobalt content [34].

In the case of Ni–Co, electrodeposition of the Co element is influenced and controlled by diffusion as compared to that of Ni which is predominantly controlled by activation. In light of this, an increase in cathodic current density results in an increase in cathodic overpotential. Concurrently, activation of the electrode reaction increases and, as a result, the rate of deposition of Ni element into the coating increases significantly [34].

In the case of Ni–Co nanocomposites, it has been reported that thinner coatings resulting from smaller amounts of electricity tend to have a higher content of nanoparticles compared to thicker coatings that are formed with larger amounts of electricity. This suggests that nanoparticles become adsorbed during the early phase of deposition and become unevenly distributed with increases in thickness of coatings [55].

### 3.3. Particle Content

**Figure 4:**
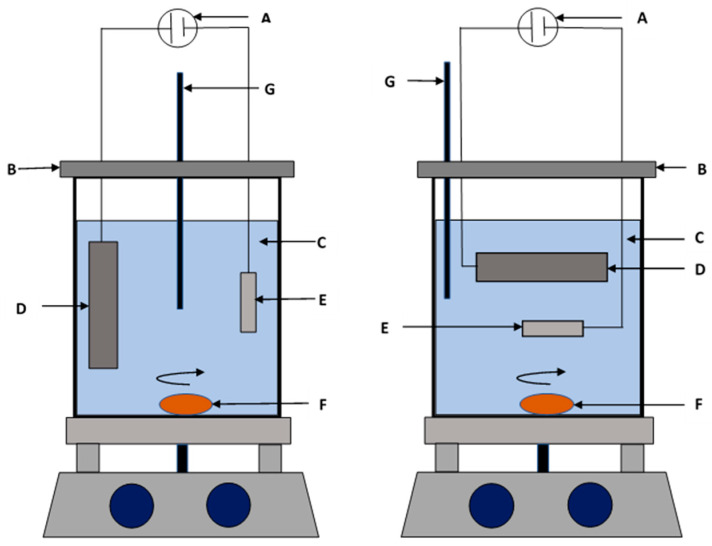
Schematic image of the deposition setups: (A) DC power supply, (B) Epoxy cover, (C) plating solution, (D) anode, (E) cathode, (F) magnetic bar and (G) external pH–temperature probe [30].

The magnetic properties, corrosion resistance, and mechanical properties of electrodeposited Ni–Co nanocomposites are mostly governed by the amount and distribution of nanoparticles. There are several interrelated factors that influence incorporation and distribution of nanoparticles into the Ni–Co coatings. These factors can be broadly classified as (i) electrolyte composition (reagents, pH, additives); (ii) nanoparticle characteristics (size, shape, type); (iii) deposition parameters (current density, bath temperature, concentration of nanoparticles and rate of electrolyte agitation); (iv) cobalt content in the coating where the Co^2+^ cations that are adsorbed on the particle surface increase the incorporation of nano particles into the coating deposits, causing an increase in nanoparticle deposition with increase in cobalt content [28,51,56]; and (v) electrode orientation, which determines the incorporation efficiency of the nanoparticles. Increase in nano particle content on Ni–Co nano composite coatings can be improved by using the sediment deposition technique (SCD) as opposed to the conventional electrodeposition technique. In the SCD technique, the electrodes immersed in the electrolyte are placed horizontally and parallel to each other. As such, electrodeposition in this technique takes advantage of gravitational pull coupled with the electrophoresis force resulting in better incorporation of nano particles [30,49]. In conventional electrodeposition, only the electrophoresis force is utilized. Figure 4 shows the electrodeposition setups depending on electrode orientation in the electrolyte. Research shows that nanoparticle content in electrodeposited Ni–Co coatings increases steadily with increase in nanoparticle concentration to a given maximum value beyond which the nanoparticle content in the deposit decreases. This increase in nanoparticle content with increase in concentration can be attributed to increased transportation of the nanoparticles to the cathode surface where more and more nanoparticles can be engulfed in the growing Ni–Co matrix. At high concentrations of nanoparticles, the interaction equilibrium between the suspended nanoparticles and the embedded ones is exceeded, beyond which the surface of the cathode becomes covered such that more suspended nanoparticles cannot be embedded into the coatings. Moreover, there is increased mechanical collisions between the nanoparticles and this reduces their transportation efficiency across the electrolyte bulk. The content of nanoparticles in the coatings therefore decreases.

### 3.4. Electrolyte Agitation

**Figure 5:**
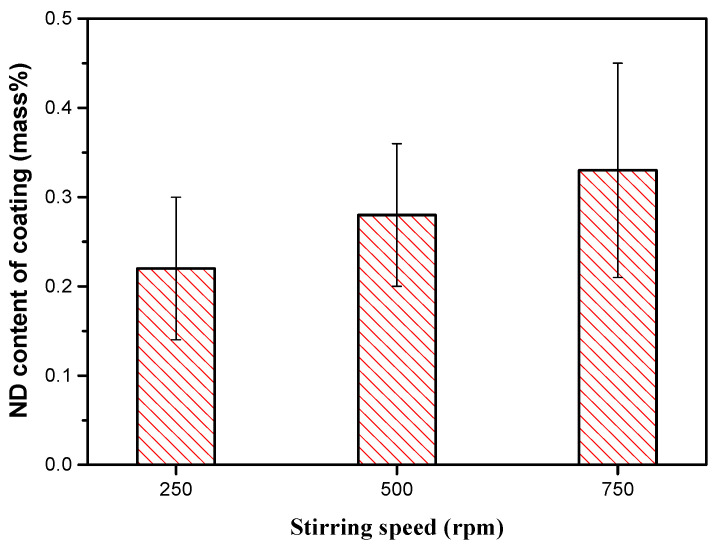
Relation between stirring speed and nano diamond (ND) content of the coatings [55].

High electrodeposition current density translates to high deposition rates, which almost always causes burrs on the cathode surface as well as increasing the coating roughness. Electrolyte agitation causes a distinct reduction of burrs that are formed on the edges of coated substrates and this reduces the coating roughness and improves uniformity [53]. Furthermore, increasing the stirring speed of the electrolyte during the deposition process increases the content of nanoparticles deposited in the Ni–Co coatings up to a given maximum level beyond which the content reduces [26,57]. At low agitation rates, the concentration of nanoparticles surrounding the cathode may reduce, resulting in the feed rate of the nanoparticles being lower than their adsorption into the Ni–Co matrix. Additionally, incomplete dispersion as a result of insufficient convection may cause agglomeration of nanoparticles and gravity settling. The surface energy of nanoparticles is greatly reduced when nanoparticles agglomerate, and this lowers the content in the deposited coatings. At higher agitation rates, the volume of nanoparticles reaching the cathode surface (mass transfer) increases thereby increasing the overall content of nanoparticles in the coatings. Goto et al. [55] reported that for the range of the experiment conducted, the nano diamond (ND) content in the coatings increased with increase in the stirring speed as shown in Figure 5. Where pulse electrodeposition is conducted using sediment deposition technique, agitation is a factor of $T_{ON}/T_{OFF}$ ratio, where $T_{ON}$ and $T_{OFF}$ represent the time intervals when the agitation is on and off. In this technique, the nanoparticles settle on the horizontal cathode with aid from gravitational force. As such, the lower the $T_{ON}/T_{OFF}$ ratio, the higher the rate of nanoparticle incorporation into the growing Ni–Co matrix [57].

Excessive rotation is however detrimental to coating quality. Vigorous hydrodynamic forces are generated at high agitation rates and these forces pluck out nanoparticles from the cathode surface before they become successfully embedded in the growing matrix surface, leading to lower nanoparticle content in the deposited coatings. This conclusion was reported by [26,58,59] who related the increase in corrosion resistance up to a maximum value to an increase in electrolyte agitation rate, beyond which it decreased with further increase in agitation rate.

### 3.5. Temperature

Shi et al. [60] postulated that there is a two-fold effect with respect to temperature. When electrolyte temperatures are low, there is an increase in nanoparticle kinetic activity with a rise in the temperature of the bath, and this boosts adsorption of nanoparticles into the metal matrix. Increasing the temperature decreases the density and viscosity of the bath, thereby improving the mobility of ions within the electrolyte. As such, coatings deposited at higher temperatures exhibit superior properties owing to thicker coatings and higher nanoparticle contents than those deposited at lower temperatures [53].

Temperature variation has also been reported to alter the size of crystals in the deposited coatings. Prabu and Wang [53] reported that increasing temperature from 20 to 60 °C resulted in larger and sharper crystals. This was attributed to increase in rate of reducible ion diffusion to the cathode with an increase in temperature which decreased the polarization resistance. Research on the effect of temperature on Ni–Co coatings shows that the deposition rate increases with increases in temperature of the electrolytic bath [4]. Idris et al. [54] reported that, for Ni–Co coatings electrodeposited using high speed jet electrodeposition, an increase in temperature from 55 to 65 °C (at 1 A/cm^2^ current density) resulted in a subsequent increase in thickness of the electrodeposited coatings from 61.4 to 71.7 µm, respectively. Similar increasing trends of the thickness with rise in temperature were observed at current densities of 0.1, 0.3 and 0.5 A/cm^2^. This can be attributed to grain growth as a result of a free growth mode of Ni resulting from the temperature rise.

When the temperature exceeds certain limits, thermodynamic ion movement is enhanced greatly, and the nanoparticle’s kinetic energy increases. As a result, less nanoparticles become adsorbed into the metal matrix. This conforms to Langmuir’s adsorption theory, where temperature increase beyond certain levels has a negative effect on nanoparticle absorbability. As a result, the electric field and the overpotential of the cathode are decreased, making it harder for nanoparticles to be embedded into the coating. As such, lower contents of nanoparticles are observed in the deposited coatings.

### 3.6. Electrolyte pH

According to past research, it can be seen that the composition and structures of Ni–Co alloys and their nanocomposites can significantly affect their physiochemical properties. The effect of pH on Ni–Co deposits is predominantly dominated by three factors [61]: (i) the acidic environment in the electrolyte dissolving newly deposited metal atoms on the cathode surface, (ii) metal hydroxide formation and adsorption on the surface of the electrode, and (iii) normal electrodeposition of metals. When the electrolyte pH is low, the newly deposited metal becomes dissolved at a faster rate and formation and adsorption of metal hydroxides becomes depressed. In this case, the electrodeposition process is mainly dominated by normal electrodeposition of metals and this results in lower Co^2+^ in the bath. At higher pH values however, the formation and adsorption of metallic hydroxides is promoted, and the newly deposited metal dissolution becomes suppressed. For higher pH values, the electrodeposition process is dominated by formation and adsorption of Co hydroxides on the cathode surface and this produces higher Co contents in deposited coatings [62,63]. Tian et al. [61] reported a gradual increase in Co content from 9.4% to 19.6% with an increase in the value of pH from 2.0 to 5.4. pH value in the electrolyte has also been observed to affect current efficiency. An increase in current efficiency from 52.1% to 81.2% was also reported with increase in pH from 2.0 to 5.4. Research linking pH value to hydrogen evolution has also been reported. Increase in pH in Ni–Co-based deposits has been associated with an increase the in hydrogen evolution rate, followed by creation of trace amounts of Ni and Co hydroxides which hinder the growth of crystals [64].

### 3.7. Pulse Frequency

Increased pulse frequency has been reported to achieve good Ni–Co films with smooth surface morphology and high microhardness, as well as better corrosion resistance of deposited coatings [5,37]. Pulse frequency has a significant influence on the morphology of the deposited coatings as well as the content of nanoparticles. At higher pulse frequencies, smaller grain sizes are obtained. Bigger grains tend to be more thermodynamically stable than smaller ones, and as such, an increase in OFF-time causes the grain size to increase [65]. Similar findings were reported by Yang and Cheng [32]. This was attributed to enhancement of the nucleation process by the SiC nanoparticles by providing electro crystallization nucleation sites and retarding crystal growth. Furthermore, the content of SiC in the Ni–Co/SiC nanocomposites increased with increasing pulse frequency. When pulse frequency levels are higher, the overpotential generated is much higher, and this provides more energy for nanoparticle adsorption into the coatings. As such, it can be concluded that higher pulse frequency offers better properties for deposited coatings as a factor of increased nanoparticle content.

### 3.8. Duty Cycle

When low duty cycles are used, the resulting coating is characterized by finer and more compact structures than if higher duty cycles had been used. It has been reported that a low duty cycle increases the microhardness and corrosion resistance of electrodeposited Ni–Co/SiC nanocomposite coatings. This can be related to the increase in SiC nanoparticle content in the coatings at lower duty cycles [32]. With the increase in duty cycle, there is a transformation of the surface morphology from a branched, acicular structure to a more nodular structure. It can therefore be concluded that lower duty cycles offer the best properties for deposited materials.

## 4. Mechanism of Ni–Co–Nanoparticle Electrodeposition

**Figure 6:**
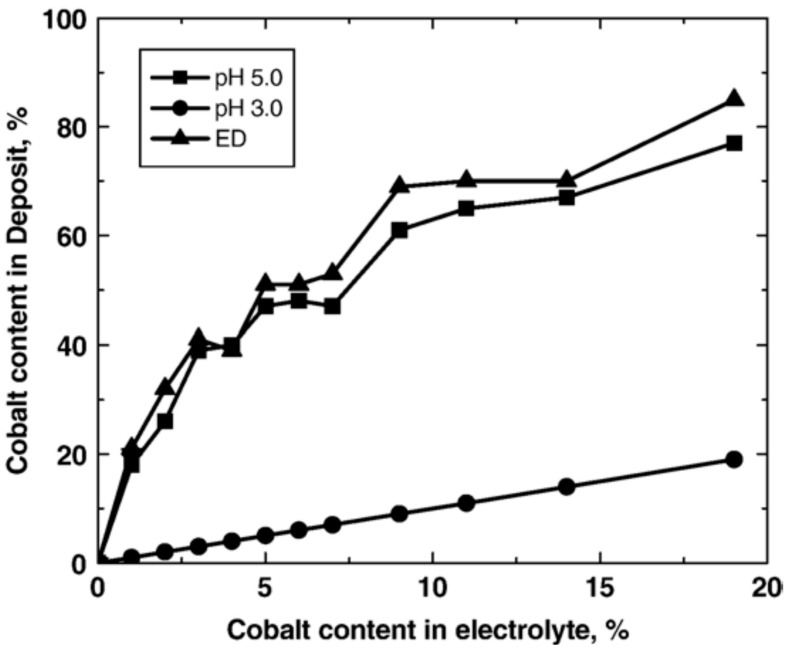
Alloy composition as a function of Co^2+^ concentration in the electrolyte [16]. Reprinted from Surface and Coatings Technology, 201, Meenu Srivastava, V. Ezhil Selvi, V.K. William Grips, K.S. Rajam, Corrosion resistance and microstructure of electrodeposited nickel-cobalt alloy coatings/Pages No. 3051–3060, Copyright (2020), with permission from Elsevier.

The process starts with complete combination of Ni and Co in the electrolyte to form a solid solution [66]. At the same time, the surfaces of nanoparticles suspended in the electrolyte adsorb positive and negative ions (particle charging). The nanoparticles are then transferred by electrophoresis force and gravitational force to the growing Ni–Co matrix where they become embedded into the deposit. Ni–Co deposition is believed to be anomalous deposition where the content of the less noble element (Cobalt in this case) is much higher than the concentration of the same element ions in the electrolyte [16,67,68]. This could be attributed to the relatively fast kinetics related to cobalt deposition. Moreover, the inhibition of nickel deposition in presence of cobalt ions is less likely as a result of evolution of metal hydroxides [67]. Figure 6 shows the cobalt content in deposited Ni–Co coatings as a function of Co concentration in the electrolyte [16].

Many models have been proposed concerning co-deposition of nanoparticles. Celis [69] suggested that it was a five-step process which took into consideration the creation of an ionic cloud that engulfed the nanoparticles, movement of the nanoparticles through the electrolyte and the diffusion layer. These steps can be classified as: (i) formation of an ionic cloud on the surface of the nanoparticles from the adsorbed ions; (ii) the charged nanoparticles are then transferred through the electrolyte bulk until they reach the hydrodynamic boundary layer; (iii) through diffusion, the nanoparticles are transferred en-masse to electrode surface; (iv) adsorption of electroactive ions and the free ions occurs on the particles on the cathode; and (v) electroreduction of adsorbed ions occurs followed by incorporation of particles into the growing metal matrix [70]. Guglielmi’s model proposed that the adsorption mechanism onto the cathode followed a two-step process for the charged nanoparticles. Firstly, the charged nanoparticles are loosely adsorbed while still being engulfed in a film of adsorbed cations [69].

## 5. Baths Used in Electrodeposition Process for Ni–Co Coating

Ni–Co alloys have been synthesized using many different electrodeposition techniques: jet, direct current and Pulse current electrodeposition techniques [5]. With the said techniques, researchers have also used different types of electrolyte baths during electrodeposition such as sulfate, sulfamate, and chloride solutions [21,22,71].

### 5.1. Chloride Baths

Many researchers over the years have used chloride baths to electrodeposit Ni–Co alloys and nanocomposites. Nickel chloride added to the chloride baths provides Cl^−^ ions which are essential for efficient dissolution of the nickel anode [72]. Fan and Piron [67] studied anomalous electrodeposition of Ni–Co alloy on Cu substrates using complex citrate and simple chloride baths. It was reported that the Ni–Co electrodeposited using the chloride baths exhibited anomalous deposition where the content of Co was higher than in the complex citrate bath. This was attributed to preferential deposition of Co which was more reactive (less noble) than Ni. It was also found that simple chloride baths were better suited at electrodepositing at current densities as low as 0.1 mA·cm^−2^.

### 5.2. Sulfate Baths

Sulfate-based baths offer good electrodeposition prospects owing to their relative affordability compared to other baths and their ability to deposit high Co content coatings [64]. These baths contain both sulfate compounds as well as Ni chloride. The Ni chloride acts an additional source of Ni^2+^ ions in the electrolyte and this influences the thickness of the deposited coatings. The Cl^−^ ions from the Ni chloride increases conductivity of the electrolyte solution by causing dissolution of Ni anodes [72,73]. Internal stresses in deposited coatings have been observed to decrease where chlorides are absent in electrolytes. Absence of Cl^−^ ions is favorable in processes where the consumable anodes remain unused. However, they are used together with other reactive agents where the anodes are consumed during the electrodeposition process [74,75].

### 5.3. Sulphamate Baths

Sulphamate and sulphamate–sulfate baths have been reported to deposit qualified and coherent Ni–Co structures [68]. For the case of high-speed electrodeposition, nickel sulphamate baths containing boric acid have been preferred over watts baths because of the ease of obtaining relatively thicker coatings with less internal tensile stresses [76]. Ni–Co coatings deposited from sulfamate–sulfate baths have been reported to have smoother and finer surfaces and this can be attributed to lower stresses being generated in coatings deposited with these baths [68].

## 6. Additives

Additives are added into the electrolyte bath during electrodeposition to increase the range of current density, change physical and mechanical properties, reduce the size of crystallites by reducing growth, increase the coating’s luster, reduce nanoparticle agglomeration, and reduce internal stresses generated during the deposition process [35].

### 6.1. Boric Acid

In 1916, Watts O.P. formulated and optimized an electrolyte comprising of nickel sulfate, nickel chloride and boric acid. This formula commonly known as Watts solution has been used extensively in nickel electrodeposition and its developmental impacts cannot be overstated [72]. The effect of boric acid in Nickel plating baths during electrodeposition has been researched over the years and the results can be summed up into six views:

(a) Boric acid suppresses oxygen evolution. Gadad and Harris [77] researched on oxygen incorporation in electrodeposited Mi, Fe and Ni–Fe alloy coatings. It was reported that an increase in applied current density resulted in an increase in the content of oxygen in Ni coatings and this posed a detrimental effect to the magnetic and electrical properties of the coatings. Addition of boric acid reduced the oxygen incorporation in all three electrodeposition systems, with less than 2 wt% oxygen observed in all cases. The buffering effect is not attributed directly to the boric acid but more to the complexing ability of boric acid with metal ions in the electrolyte.
(b) Boric acid promotes deposition of Nickel by acting as a catalyst. The adsorptive interaction of boric acid has also been observed in Ni–Zn alloy coatings where boric acid increased the current efficiency of the system at lower Zn (II) concentrations and increased the Ni content of the coatings at higher Zn (II) concentrations [78]. Significant change in primary nucleation rate coupled with suppressed secondary nucleation on coatings was also reported. Cyclic voltammetric deposition results have shown that the hydrogen evolution rate (HER) increases relative to the increase in boric acid concentration.
(c) Boric acid as a pH buffer. In electrodeposition, the practical buffer range is given at pKa ± 1, but this value is much higher in the case of boric acid (9.23 ± 1 at 25 °C). This is an anomaly considering the pH of the Ni electrolyte is 4.0. The anomaly can be attributed to formation of weak bound complexes between nickel ions and boric acid, such that the said complexes act as pH buffers [79,80]. The presence of these complexes however, has yet to be confirmed experimentally. This pH buffering phenomena has been found to be significantly influenced by the applied current density. Tsuru et al. [76] reported that at lower current densities (below 1.0 A dm^−2^), the pH buffering properties of boric acid were exhibited.
(d) Suppression of hydrogen evolution by boric acid. During electrodeposition, electric current flowing through the system causes an increase in pH and as a result, hydrogen gas is produced at the cathode. Hydrogen evolution at the cathode is detrimental to the reduction of metal ions, and therefore boric acid is added into the plating bath solution to prevent electrode surface passivation as well as act as a surface agent which acts as a selective membrane to block passage of the reduction of Nickel, while permitting the reduction of iron in a retarded state. Improving the electrodeposition current density range thereby minimizes the effect [81]. Yin et al. [82] suggested that boric acid acted like a surfactant which was adsorbed onto the surface and hinders hydrogen evolution. The adsorbed boric acid interferes with the alloy nucleation process thereby reducing the hydrogen evolution rate in Ni-enriched phases [82]. It should be noted that the hydrogen evolution suppressing properties of boric acid have only been observed in presence of nickel ions and this suggests that there exists a mutual interaction between nickel and boric acid [76].
(e) Reduction of passive film formation by boric acid during Ni electrodeposition. Boric acid was found to significantly deter surface passivation on Ni reduction in Fe–Ni setups [82]. Tsuru et al. [76] suggested that, by acting as a surface agent, boric acid hindered passivation of the electrode surface during reduction of nickel.
(f) Accelerating growth rates of deposits. Boric acid improves the lateral as well as the outward growth rate during deposition of nickel [83].

It has also been reported that coatings electrodeposited in electrolytes containing boric acid exhibit better appearance coupled with reduced brittleness [81].

### 6.2. Surfactants

Nanoparticle stabilization in the electrolyte and non-agglomerated nanoparticle dispersion are key elements of producing Ni–Co nanocomposite coatings with excellent properties. Surfactants such as cetyltrimethylammonium bromide (CTAB) [84,85,86], sodium dodecyl sulfate (SDS) [87,88], and sodium lauryl sulfate (SLS) [89] are added into the Ni–Co nanocomposite coating baths to prevent nanoparticle agglomeration by reducing the electrolyte’s surface tension to create smaller hydrogen bubbles thereby limiting pitting effect [35]. Surfactants of cationic and anionic nature are commonly used owing to their significant influence on ceramic-based nanoparticle dispersibility. Surfactants added into the bath work by changing the cathode polarization potentials, thereby changing the adhesion, smoothness, grain growth rate, and grain size of the deposited coatings [90]. Several researchers have used optimum quantities of different surfactants and reported improvement in corrosion resistance and mechanical properties [91].

Different surfactants have different effects on different nanoparticles. It was reported that CTAB surfactant offered a better alternative over SDS and triton X when they were compared in deposition of SiC nanoparticles using the PRC technique [92] and the PC technique [35]. However, the advantage offered by surfactant addition is not limitless. When the concentration of a surfactant exceeds a certain limit, it becomes counter-productive. Ger M [93] observed that excessive CTAB surfactant increased nanoparticle’s adhesive force which resulted in deposition of coarser SiC nanoparticles.

### 6.3. Saccharin

Addition of saccharin results in an increase in alloy deposition overvoltage which promotes deposition of Ni while hindering that of Co, and as such the resulting Ni–Co coatings exhibit reduced Co content [4,94]. Research shows that surface morphologies of Ni–Co coatings exhibited colony-like morphologies which consist of grain morphologies where several grain colonies converge to form one larger colony. As such, grain size is greatly reduced owing to the grain refinement phenomena. This is achieved by inhibition of pyramidal growth by saccharin thereby leading to the production of shiny, smooth surfaces [94]. This concurs with Weil and Cook [95] who reported that addition of organic additives such as coumarin and thiourea into the Ni–Co electrolytes hindered the growth of pyramids, caused surface roughness reduction, grain size reduction and increased surface brightness.

Increase in saccharin content in the coatings up to a certain value also improves the microhardness of Ni–Co coatings beyond which the microhardness reduces with increase in saccharin content. Li et al. [94] reported that increase in saccharin content to 3 g/L, 4 g/L and 5 g/L resulted in increased microhardness values of 456 kg/mm^2^, 507 kg/mm^2^ and 554 kg/mm^2^, respectively. This conclusion was also reached by Wang et al. [96]. The increase in microhardness with increase in saccharin content to a certain value can be attributed to grain refining effect, and the decrease in microhardness beyond that level of saccharin can be attributed to the inverse Hall–Petch relationship when refining of grain size reaches a certain level [97]. The drawback to adding saccharin to the electrolyte is the reduced ductility of the resulting Ni–Co coatings owing to the sulphur and carbon impurities that are usually present in saccharin laden nanocrystalline coatings. The said impurities separate into grain boundaries thereby preventing the efficient sliding of grain boundaries and hence the low ductility [98].

Saccharin has been reported to act as an internal stress reliever in electrodeposited Ni–Co coatings and this has been attributed to grain refinement. Internal stresses are developed within the coating layer during the electrodeposition process and they cause oriented resultant strain in deposited coatings. Hydrogen ion reduction occurs at the cathode, and the small sized H^+^ promote favorable conditions for diffusion to the coating’s active centers. As the H^+^ become transformed into H molecules, internal stresses are developed as the volume changes. They are classified into three main categories [99,100,101,102]:

(i) Macroscopic stresses. These are caused by inhomogeneity in the deposited coatings. These comprise of either compressive or tensile stresses and they occur in galvanic cells.
(ii) Microscopic stresses. These originate at grain boundaries and at locations where dislocations accumulate.
(iii) Sub-microscopic stresses.

While internal stresses have been known to improve hardness and abrasion resistance of deposited coatings, at high levels, these stresses increase the coating’s brittleness. Brittle coatings develop extensive microcracks which expose the substrate surface to corrosive attack and degradation when exposed to a corrosive medium. The saccharin molecules are reversibly adsorbed on active sites thereby hindering growth of crystals and impeding surface diffusion of adatoms. As such the volume of grain boundaries increases and this dissipates the energy created by internal stresses [103].

Saccharin has been employed in tensile research of electrodeposited Ni–Co nanocomposite coatings. Wang et al. [96] reported that PC deposited Ni–Co/Al_2_O_3_ nanocomposites containing saccharin exhibited low-temperature superplasticity, where a maximum elongation of 632% was achieved at a strain of 1.67 × 10^−3^ s^−1^ and a temperature of 823 K. The dominant superplastic accommodation process was taken to be dislocation glide.

## 7. Properties of Electrodeposited Ni–Co Coatings

### 7.1. Microstructure

**Figure 7:**
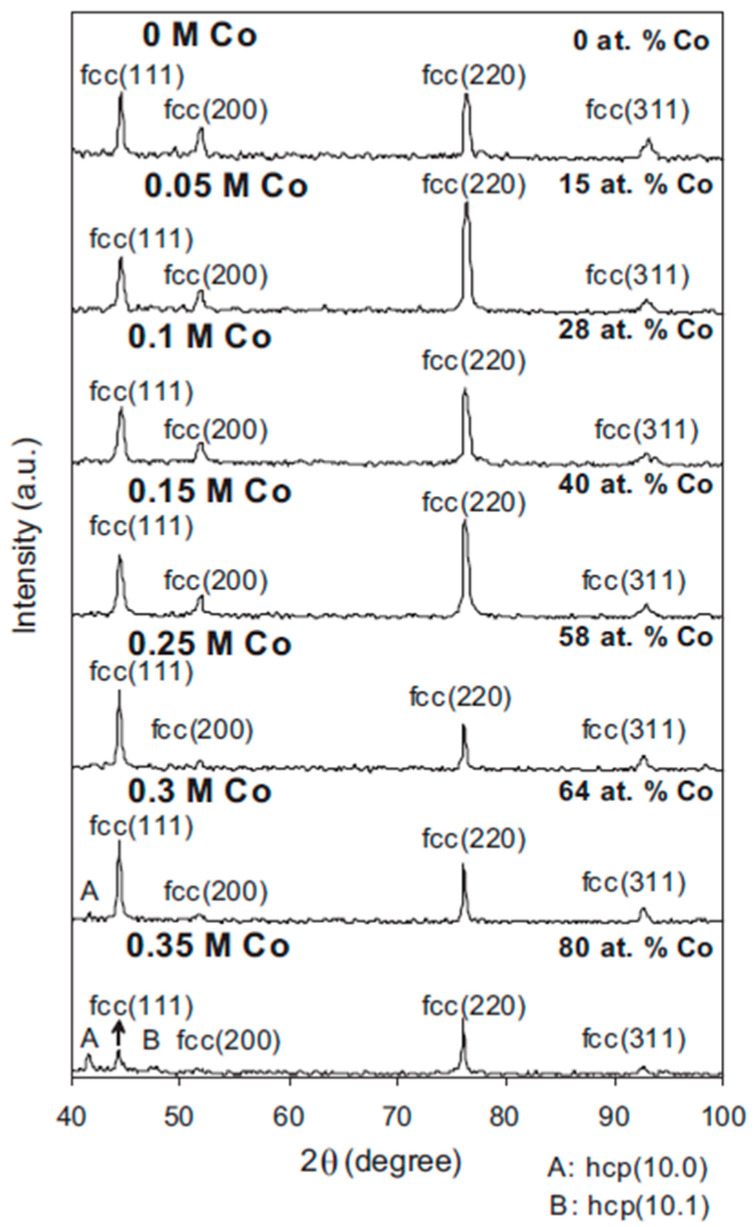
X-ray diffraction (XRD) patterns of Ni–Co films deposited from the electrolytes containing different Co concentrations [68]. Reprinted from Applied Surface Science, 258, Ali Karpuz, Hakan Kockar, Mursel Alper, Oznur Karaagac, Murside Haciismailoglu, Electrodeposited Ni–Co films from electrolytes with different Co contents/Pages No. 4005–4010, Copyright (2020), with permission from Elsevier.

As stated earlier, Ni–Co coatings deposition is anomalous. This can be explained as a function of local pH increase resulting in creation and adsorption of metallic hydroxides, as well as a faster rate of cobalt hydroxide adsorption [104], deposition of Co cations (first step) followed by Ni deposition (second step) in a two-step process [105], and preferential Co element deposition which causes the diffusion layer to become depleted [4]. Ni–Co alloys with up to 58 wt% cobalt content exhibit the single phase of Ni matrix with FCC type of phase structure. When the cobalt content is in the range between 64 wt% to 80 wt% the phase structure becomes a combination of FCC and HCP as shown in Figure 7 [68].

**Figure 8:**
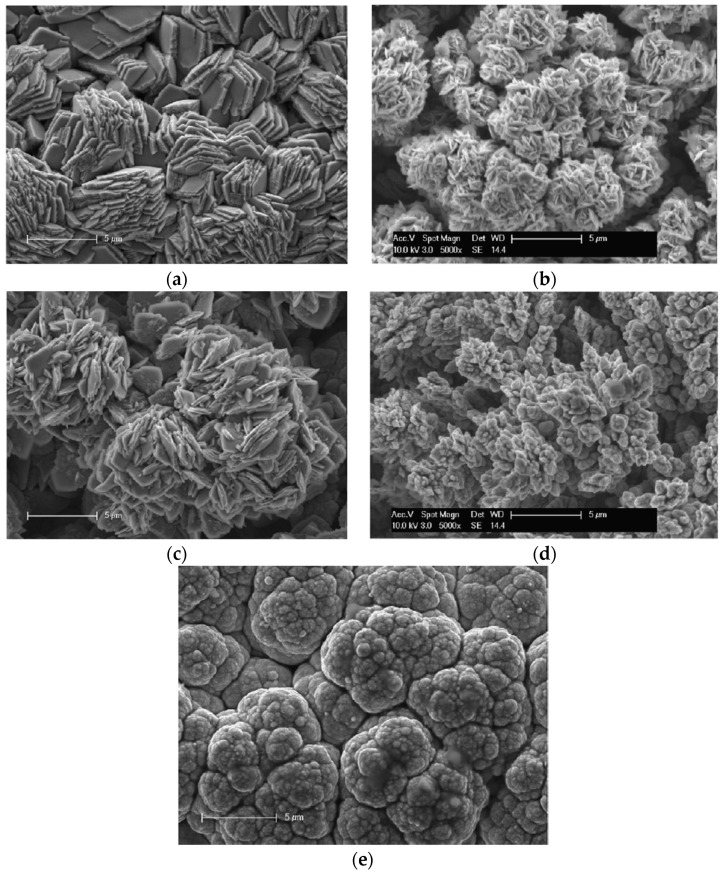
SEM micrographs of Ni–Co deposits obtained at a current density 65 mA cm^−2^ from an electrolyte with different Ni^2+^/Co^2+^ concentration ratio: (**a**) 0.25, (**b**) 0.5, (**c**) 1, (**d**) 2 and (**e**) 4 [106]. Reprinted from Materials Chemistry and Physics, 120, L.D. Rafailovic, H.P. Karnthaler, T. Trisovic, D.M. Minic, Microstructure and mechanical properties of disperse Ni–Co alloys electrodeposited on Cu substrates/Pages No. 409–416, Copyright (2020), with permission from Elsevier.

Ni–Co alloys with Co content above 80 wt% exhibit complete HCP phase structure [16,40,68]. Ni–Co alloy surface morphologies are significantly influenced by the coating’s chemical composition. Rafailovic et al. [106] researched the mechanical properties of Ni–Co alloys deposited on Cu substrates. It was reported that a platelet structured morphology was formed for coatings with a Ni^2+^/Co^2+^ ratio of 0.25 at 65 mA cm^−2^. The surface morphology exhibited enhanced dendritic growth when the ratios were 0.5 and 2. At the highest Ni^2+^/Co^2+^ ratio of 4, the surface morphology exhibited cauliflower structure as shown in Figure 8.

The microstructure of electrodeposited Ni–Co-based coatings is affected by evolution of hydrogen gas at the cathode. Hydrogen is a by-product of the electrodeposition process owing to the breakdown of water molecules in the plating solution during the electrodeposition process. This holds both an advantageous and detrimental effect depending on the desired output of the process. Hydrogen offers a promising versatile, efficient, and clean candidate for use as an energy source to replace commonly used fossil fuels which cause CO_2_ emissions that are harmful to the environment [107,108,109]. Hydrogen can be successfully generated using the less efficient (higher operating cost) alkaline water electrolysis [110,111], or using low hydrogen evolution reaction (HER) overpotential electrodes [112]. Ni-based alloys and compounds form such electrode materials owing to their low cost coupled with high catalytic activity and stability [113]. In the case of depositing quality coatings however, the evolution of hydrogen gas is detrimental to the structure, hence the critical need to control its production. The synthesized hydrogen gas attaches to the surface of the base metal creating a blanket of air that inhibits nucleation and deposition of the coatings and this causes poor adherence leading to non-uniform coatings [114]. The hydrogen evolution phenomenon has been reported to be more significant at higher current densities, owing to lower hydrogen overpotential where numerous gas pits are formed on the coating surface as a result of the hydrogen produced [112].

### 7.2. Mechanical Properties

**Figure 9:**
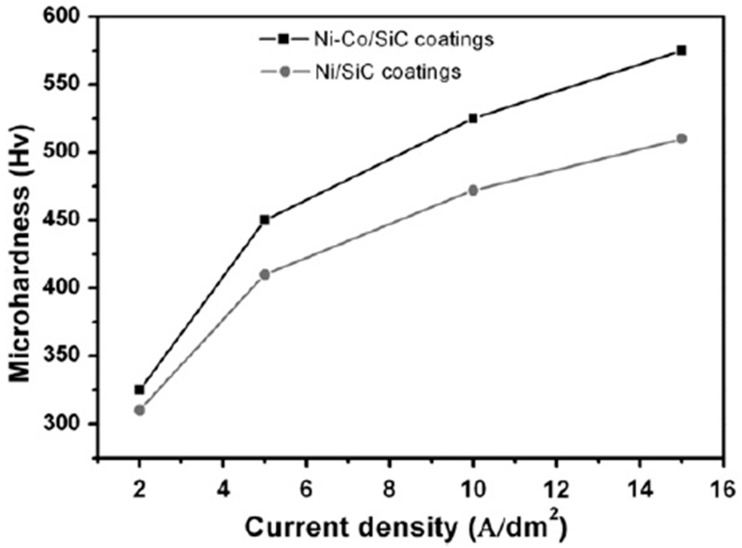
The effect of current density on microhardness of Ni–Co/SiC and Ni/SiC coatings [115]. Reprinted from Surface and Coatings Technology, 206, S.M. Lari Baghal, M. Heydarzadeh Sohi, A. Amadeh, A functionally gradient nano-Ni–Co/SiC composite coating on aluminum and its tribological properties/Pages No. 4032–4039, Copyright (2020), with permission from Elsevier.

Microhardness in electrodeposited Ni–Co coatings increases with an increase in Co content in the coatings. Baghal et al. [115] reported similar findings when the microhardness of Ni–SiC coatings was compared to Ni–Co/SiC coatings as a function of increasing current density, as shown in Figure 9.

Enhancement of micro-hardness in Ni–Co alloy coatings as a function of Co content can be linked to the (i) grain size reduction, (ii) phase composition, where two-phase structures are formed, and (iii) solid solution strengthening [16,20,116]. Babak [12] reported similar observations where coatings up to 45 wt·% Co were characterized solely by FCC lattices. No other phase was observed from the XRD pictographs and it was suggested, therefore, that the effect of phase composition on microhardness of the said coatings was not significant. This was held as evidence that solid solution hardening played a key role in the microhardness of the deposited Ni–Co coatings. The grain size of deposited Ni–Co alloy coatings decreased gradually with increase in content of Co element in the coatings [12].

**Figure 10:**
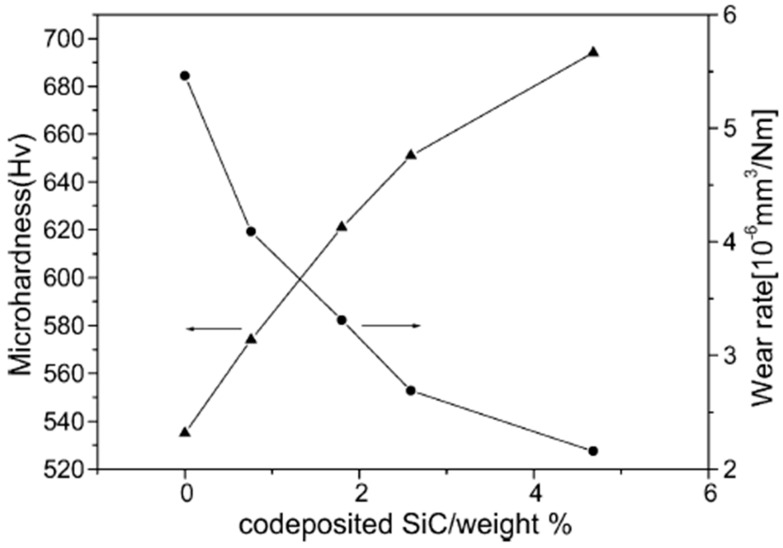
Microhardness and wear rate of the nanocomposite coating vs. weight percentage of co-deposited SiC particulates in the nanocomposite coating [60]. Reprinted from Applied Surface Science, 252, Lei Shi, Chufeng Sun, Ping Gao, Feng Zhou, Weimin Liu, Mechanical properties and wear and corrosion resistance of electrodeposited Ni–Co/SiC nanocomposite coating/Pages No. 3591–3599, Copyright (2020), with permission from Elsevier.

The wear rate of deposited Ni–Co coatings has been observed to decrease with increase in Co content in the deposits. The phenomena can be associated with increase in microhardness with increase in Co content. A relationship exists between wear rate and microhardness of a coating, called Archard’s law, which provides that the sliding wear volume loss is directly proportional to friction coefficient and inversely proportional to the hardness of the material [117]. This relationship can be seen in Figure 10 [60].

**Figure 11:**
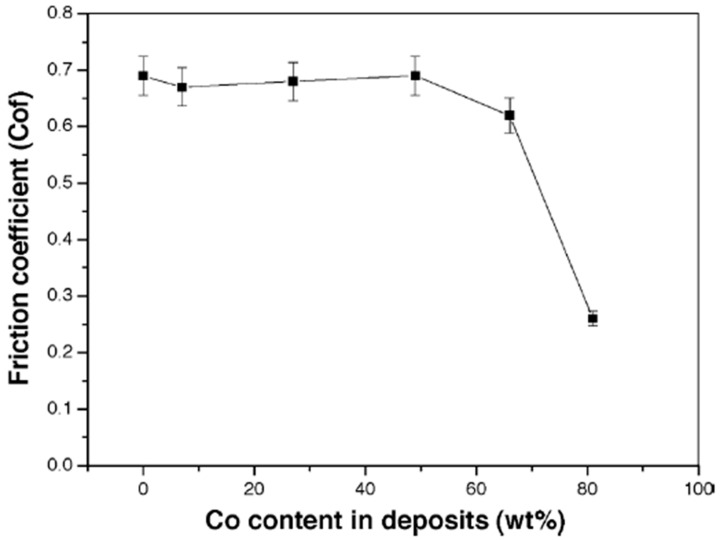
Friction coefficient as function of Co content in the Ni–Co alloy deposit [20]. Reprinted from Applied Surface Science, 242, Liping Wang, Yan Gao, Qunji Xue, Huiwen Liu, Tao Xu, Microstructure and tribological properties of electrodeposited Ni–Co alloy deposits/Pages No. 326–332, Copyright (2020), with permission from Elsevier.

In some instances, however, the wear rate decreases with decrease in microhardness, a deviation from Archard’s Law. This is concurrent with findings reported by Wang et al. [20] where the decrease in wear rate beyond 49 wt% with a concurrent decrease in microhardness from 462 HV to 298 HV was attributed to changes in the phase structure. As Co content increases, the phase structure of the deposited Ni–Co coatings changes from solely FCC structure, to FCC coupled with HCP structure, and when the Co content goes beyond 80 wt% (See Figure 7), the phase structure is transformed to a predominantly HCP structure. This transformation in phase structure to a higher ratio of HCP causes a decrease in the coefficient of friction (COF) of the deposited Co-rich coatings and therefore decreased wear loss [20]. As such, the decreasing wear rate was associated with the decreasing coefficient of friction (COF). This is shown in Figure 11.

Magnetic measurements done on electrodeposited Ni–Co coatings show that Co content has significant influence on the magnetic and structural properties of the coatings. Increase in Co content in the coatings results in a gradual increase in the saturation magnetization. This conclusion is concurrent with Karpuz et al. [68] where the highest in-plane saturation magnetization of 1000 emu/cm^3^ was achieved at the highest contents of Co (80%). The same trend was also reported by [118].

**Figure 12:**
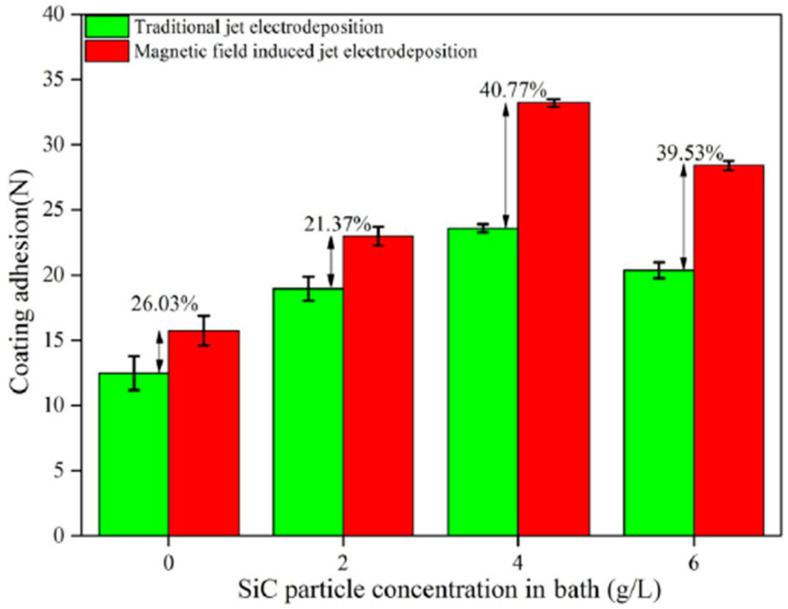
Adhesion of the composite coatings [120]. Reprinted from Journal of Alloys and Compounds, 791, Wei Jiang, Lida Shen, Mingyang Xu, Zhanwen Wang, Zongjun Tian, Mechanical properties and corrosion resistance of Ni–Co–SiC composite coatings by magnetic field-induced jet electrodeposition/Pages No. 847–855, Copyright (2020), with permission from Elsevier.

Coatings improve the performance of the component by isolating the material’s structure from the environment. Different substrate materials have been used for deposition of Ni, Ni–Co, and Ni–Co-nanocomposite based coatings ranging from steel, aluminum, to copper [106,115]. Failure of systems is usually associated with substrate–coating interface failure owing to the differences in mechanical and physical properties. Coating adhesion plays a key role in a surface’s wear resistance and it is measured through friction testing where the friction abruptly changes when the coating breaks, also known as the critical load point. As such, a larger critical load indicates stronger coating adhesion [119]. Different mechanisms aimed at improving the critical load have been researched over the years. Wei et al. [120] reported that use of the magnetic jet electrodeposition technique (MJE) yielded a higher adhesion compared to traditional jet electrodeposition technique (TJE). At 4 g/L, TJE technique had a maximum adhesion of 23.58 N compared to 33.20 N under MJE technique as shown in Figure 12.

The adhesion of Ni–Co binary alloys can be further improved by incorporating nanoparticles into the matrix such that the effective area of contact between the substrate and coatings is increased, and the dispersion strengthening mechanism of the nanoparticles improves the coating’s adhesion [121,122]. Another concept that has been considered for adhesion improvement is functionally graded materials (FGMs), whereby interfacial problems are mitigated by controlling progressive changes in structure and properties [123].

Ni–Co-nanocomposite coatings deposited at high current densities have exhibited higher surface roughness. This can be traced to adsorption of nanoparticles into the coating surface coupled with formation of pits and crevices as a result of an increase in hydrogen evolution rate at high current densities. Similar observations were made by Dheeraj et al. [35] where a surface roughness of 2.31 ± 1.78 μm was reported for sample S3 which represented coatings deposited at higher current densities.

### 7.3. Corrosion Behaviour

**Figure 13:**
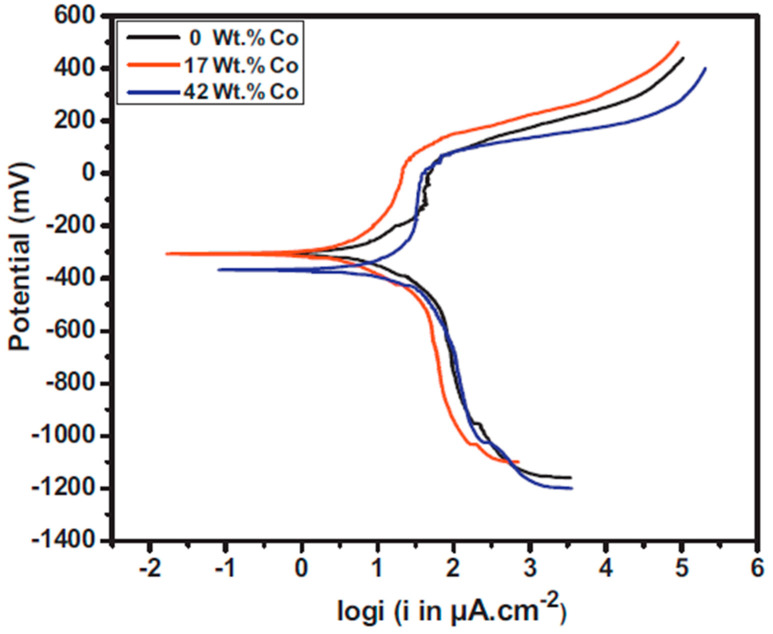
The potentiodynamic polarization curves of the Ni–Co alloy coating as a function of the cobalt content [126]. Reprinted from Applied Surface Science, 307, Babak Bakhit, Alireza Akbari, Farzad Nasirpouri, Mir Ghasem Hosseini, Corrosion resistance of Ni–Co alloy and Ni–Co/SiC nanocomposite coatings electrodeposited by sediment codeposition technique/Pages No. 351–359, Copyright (2020), with permission from Elsevier.

The corrosion behavior of electrodeposited Ni–Co alloy can be attributed to: chemical composition, grain size, preferred orientation, and phase structure. In the case of chemical composition, it has been reported that formation of Ni–Co alloy can change the nobility of the materials thereby affecting corrosion resistance [124]. Co is less noble that Ni, that is, it is more reactive compared to Ni. Therefore, increasing the Co content is bound to produce coatings with greater electrochemical activity than that of purely Ni coatings [125]. The polarization resistance of Ni–Co alloys has been reported to increase with increase in Co content up to a given limiting value, beyond which the corrosion resistance decreases with further increase in Co content. This is concurrent with findings reported by Babak et al. [126] where Ni–17Co coatings exhibited better corrosion resistance (10.08 kΩ cm^2^) compared to Ni–42Co alloy coatings (3.32 kΩ·cm^2^) as shown in Figure 13.

The phase structures of Ni–Co binary alloy coatings have been observed to consist of FCC single-phase solid solutions [12,126]. In the case of the coating’s materials microstructure, single phase structures have proven more corrosion resistant than two-phase structures. Corrosion attacks usually occur along grain boundaries (between phases) owing to the galvanic cells that are created between the phases and their higher levels of energy compared to parts located within the crystal itself. Moreover, base centered cubic (bcc) phases have lower corrosion resistance compared to face centered cubic (FCC) phases as a result of their lower packing factor.

As a factor of preferred orientation, it has been reported that Zn–Ni alloys exhibit high corrosion resistance owing to crystallographic planes being predominantly present with higher packing densities [127]. Babak et al. reported that Ni–17Co alloy coatings exhibited high corrosion resistance as a function of predominantly (111) preferred orientation. Lupi et al. [66] found that electrodeposited Ni–Co alloys containing 40%–50% Co content provide the best catalytic properties of the alloy for the reaction leading to evolution of hydrogen in alkaline media.

## 8. NiCo–Ceramic Composites

Nanoparticles have been added to the Ni–Co matrix to further improve the properties. Ni–Co nanocomposite coatings exhibit superior hardness and wear resistance to alloy coatings. This can be attributed to inherent hardness of nanoparticles used, coupled with nanoparticle strengthening through Orowan mechanism [30]. According to this mechanism, the adsorbed nanoparticles hinder the formation and propagation of dislocations as the metallic matrix carries the load.

In some instances, the increase in Co concentration in the bath has been reported to improve nanoparticle incorporation thereby improving coating properties such as corrosion resistance [30]. This effect of nanoparticles can be linked to several factors:

(i) Reduction of exposed area open to corrosive media. Nanoparticles used in Ni–Co nanocomposite electrodeposition are usually ceramics. When these nanoparticles are uniformly distributed in the Ni–Co matrix, they minimize the metallic area that is exposed to corrosive attacks and, as a result, the corrosion potential is shifted to nobler values [128].
(ii) SiC nanoparticles acting as physical barriers that hinder creation and propagation of corrosive pits.
(iii) Nanosized SiC particles which offer greater corrosion resistance than micro-sized particles when used to deposit Ni–Co/SiC nanocomposites. Owing to their smaller sizes, such nanoparticles can access structural defects such as porosities and cracks thereby mitigating the corrosive effect at such locations.
(iv) Formation of micro-galvanic cells. The metallic matrix acts as an anode while the nanoparticles act as cathodes when the Ni–Co nanocomposite coatings are exposed to corrosive media. Where the metallic matrix’s electrochemical potential is less positive than that of the nanoparticles, the corrosion mechanism of the micro–galvanic cells is transformed to uniform corrosion from pitting and localized corrosion [126].

Other factors that may be associated with metallic alloy nanocomposite coatings include: reinforcing phase induced hardening, texture evolution, grain refinement of the matrix, and solid solution strengthening depending on selected matrix [129,130,131]. Wear resistance of Ni–Co alloy coating is greatly improved by incorporation of nanoparticles in the matrix. This increase can be attributed to the dispersion strengthening effect of adsorbed hard nanoparticles coupled with grain-refining tendencies. The uniform dispersion in the matrix and to a small extent particle agglomeration may be linked to improved wear resistance in Ni–Co nanocomposites. Similar conclusions were reached by Shi et al. [60].

### 8.1. Effect of Al_2_O_3_ Nanoparticles

Al_2_O_3_ nanoparticles have inherent high corrosion resistance, and in the nano-scale range, can efficiently contribute to porosity reduction, thereby further boosting the corrosion resistance of deposited coatings [132,133]. Incorporation of Al_2_O_3_ nanoparticles into the Ni–Co matrix produces nanocomposite coatings characterized by nodular, uniform, and compact morphology [26]. The content of Al_2_O_3_ in the coatings increases with increases in Al_2_O_3_ concentration in the electrolyte. The same trend was reported by Borkar [31], with a maximum Al_2_O_3_ content being achieved with 40 g L^−1^ nanoparticle concentration, beyond which the content decreased. Ni–Co alloy coatings exhibit a face centered cubic (FCC) crystal structure and the same has been reported for Ni–Co/Al_2_O_3_. However, the crystal orientation of the resulting Ni–Co/Al_2_O_3_ nanocomposite coating undergoes transformation from crystal face (200) lattice to (111) lattice [40].

Like with most Ni–Co nanocomposite coatings, adsorption of Al_2_O_3_ nanoparticles into the Ni–Co matrix results in a subsequent increase in the coating’s hardness and wear to a certain maximum, beyond which the coating becomes brittle and spalls off. Tian [26] concluded that an increase in Al_2_O_3_ nanoparticle concentration in the bath caused a subsequent increase in the corrosion resistance of the Ni–Co/Al_2_O_3_ nanocomposite coatings up to a certain limiting value, suggesting that optimal operating conditions and parameters are key to corrosion resistance maximization. This may be attributed to uniform dispersion of Al_2_O_3_ nanoparticles in the Ni–Co matrix.

It has been suggested that Al_2_O_3_ nanoparticles may also improve deposition of Ni and Co elements in the coatings [26]. Wear resistance is also higher for Ni–Co/Al_2_O_3_ compared to their alloy counterparts and this too increases with increases in nanoparticle content.

### 8.2. Effect of SiC Nanoparticles

Ni–Co/SiC nanocomposite coatings exhibit a porous free and dense microstructure characterized by uniformly distributed SiC nanoparticles throughout the deposited coating surface [126]. The phase structure of Ni–Co/SiC nanocomposites is predominantly face centered cubic. Silicon carbide (SiC) is a chemically inert semi-conductor material [134]. Embedding of SiC nanoparticles causes a significant improvement in the corrosion resistance, wear resistance and microhardness of the deposited coatings [31]. Babak [126] reported the same findings, with the highest corrosion resistance achieved with coatings containing 8.1 vol.% SiC nanoparticles.

In the case of Ni–Co/SiC nanocomposite coatings, it has been observed that the concentration of Co element in the electrolyte has a significant effect on SiC content, whereby SiC content increases considerably with increasing Co element concentration. Babak [135] reported that SiC content increased from 2.0 vol.% to 8.1 vol.% with increases in the concentration of Co in the electrolyte. This phenomenon was attributed to ease of Co^2+^ cations adsorbing on nanoparticle surfaces compared to Ni^2+^ cations. Therefore, there was an increase in the adsorbed positive charge on the surface of the SiC nanoparticles as the Co element concentration increased. Generally, mass transfer of positively charged SiC nanoparticles towards the cathode surface is enhanced since there is an increase in the electrophoresis force exerted on them [28]. However, when the metallic cations become saturated on the SiC nanoparticle surface, a decrease in SiC content with increasing Co element concentration is observed.

Wear rate has been reported to increase with increases in SiC nanoparticle content in Ni–Co matrices [60]. As more content of SiC nanoparticles is added into the coatings, the grain refining and dispersion strengthening effects become magnified thereby improving the wear resistance of the nanocomposite coatings.

### 8.3. Effect of ZrO_2_ Nanoparticles

ZrO_2_ nanoparticles are known for their fracture toughness, stress induced transformation, and strength [136,137,138]. As such, they are an important consideration in nanocomposite coating deposition for wear protection in environments at high pressure and high temperature. Ni–Co/ZrO_2_ nanocomposite coatings also exhibit high hardness and excellent corrosion resistance.

## 9. Applications

Electrodeposited Ni–Co alloys and nanocomposites exhibit unique properties and, as such, they are used for a wide variety of industrial applications. Their combined reduced localized corrosion and microhardness greatly improves protective coating performance. These coatings can therefore be used to protect less wear resistant and softer substrate surfaces for use in industry.

Karpuz et al. [68] reported that Ni–Co coatings electrodeposited from baths containing nickel sulfamate, boric acid and cobalt sulfate have potential for application in magnetic sensors. These magnetic properties of deposited Ni–Co coatings and their nanocomposites offers attractive potential to serve as soft magnets for motors, power supplies and high-efficiency transformers [3].

Ni–Co hydroxide nanosheets have been identified as candidates for pseudocapacitor application to meet the ever-growing demand for new energy storage devices. Pu J, et al. [139] researched on Ni–Co layered double hydroxides (LDHs) nanosheets and reported that the nanosheets exhibited excellent specific capacitance of 1734 F g^−1^ at 6 A g^−1^. The nanosheets also exhibited better stability with a capacitance retention of 86% in the galvanostatic charge–discharge test after 1000 cycles.

## 10. Future Scope and Recommendations

Key areas that have been identified for future additional research include [140,141]:

(i) Corrosion resistance behavior in varying environments, such as steady and dynamic conditions.
(ii) Tribological properties such as dry and wet abrasive behavior under controlled loads.
(iii) Electroless deposition of Ni–Co coatings, which offers a more competitive and specialized option.
(iv) Thermal oxidation resistance of Ni–Co alloy matrices.
(v) More efficient electrolyte agitation techniques such as submerged jet impingement and flow cells, jet eductors, and ultrasound.
(vi) Extensive research on hydrogen evolution mitigation in electrodeposited Ni-based coatings by using pulse electrodeposition and additives.
(vii) Use of response surface methodology to optimize the Ni–Co electrodeposition process and increase accuracy of the desired properties and also predict tested properties.

Hydrogen evolution affects the coating structure of deposited coatings. Several approaches have been used with different materials to great success. Kannan and Wallipa [114] coated a magnesium alloy with calcium phosphate using constant-potential and pulse-potential methods and analyzed the in vitro corrosion resistance properties. It was reported that the polarization resistance of pulse-potential deposited coatings was three times higher than that exhibited by constant-potential deposited coatings, and this was associated with the calcium phosphate particles being closely packed for pulse-potential coatings. This provides an interesting approach that can be researched on using Ni and Ni–Co based coatings.

Several other hypotheses have been postulated for the mitigation of hydrogen evolution in the electrodeposition process in different coatings. In past research, an organic solvent (ethanol) was added to the electrolyte bath to slow down hydrogen evolution on a magnesium alloy by decreasing conductivity of the plating solution, resulting in decreased hydrogen bubble bursting rate and hence a highly dense coating was deposited [142]. This approach of slowing down hydrogen evolution by decreasing the conductivity, however, results in lower deposition rates. Metal deposition utilizes the OH^−^ ions generated during H_2_O breakdown whereby the metallic ions are reduced to form the metal. The mechanisms for hydrogen generation and metal-hydroxyl ion adsorption are shown in Equations (14)–(18) [143,144].

(14) $$2H_{2}O+2e^{-}=H_{2}+2{\mathrm{OH}}^{-}$$

(15) $$2H+2e^{-}=H_{2}$$

(16) $$M^{2+}+{\mathrm{OH}}^{-}=M{\left(\mathrm{OH}\right)}^{+}$$

(17) $$M{\left(\mathrm{OH}\right)}^{+}\to M{\left(\mathrm{OH}\right)}_{\mathrm{ads}}^{+}$$

(18) $$M{\left(\mathrm{OH}\right)}_{\mathrm{ads}}^{+}+2e^{-}=M+{\mathrm{OH}}^{-}$$

where M can be Ni or Co ions. As such, a balance must be struck between the rate of hydrogen evolution and the deposition rate, and this offers an interesting area for research and application in Ni and Ni–Co based electrodeposited coatings. Other additives used include polyethylene glycol and di-sodium ethylenediamine tetraacetic acid (EDTA) in pulse copper deposition, and it has been reported that this improves the throwing power, current efficiency, and thickness of deposited coatings [145]. These additives can also be considered for electrodeposited Ni and Ni–Co based coatings.

It is advisable to use larger sample sizes because they hinder the manifestation of edge effects that are common in smaller sample sizes. This is especially common where the current density used is high with respect to the substrate’s surface area.

The adhesive force that exists between the substrate and the coatings plays a major role in the wear resistance of the material. As such, ensuring a good bond exists between the deposit and substrate surface is key. Copper electrodes tend to exhibit better adhesive properties compared to their steel counterparts, but they are also more costly. Using a pre-treatment step offers the chance to improve this bond, especially where the substrate is made of steel. From personal experience, a triple immersion procedure of electronic cleaning can be used for better adhesion. This comprises of degreasing using electro-hydrostatic fluid, then removal of oxide layer by passing the substrates through a strong activating solution, and finally removal of carbon-black by passing the substrates through a weak activating solution [49]. A similar pre-treatment process has been used in other coatings like Ni–W nanocomposite coatings with good adhesion translating to superb wear resistance achieved [146]. This pre-treatment process provides interesting possibilities for future use in Ni–Co alloys and nanocomposites. De-ionized water should be used to clean the substrate surface after each pre-treatment step.

Research shows that orientation of electrodes in the electrolyte plays a major role in the deposition process. Results obtained from Ni–Co deposition suggest that the sediment deposition technique (SCD) is more favorable compared to conventional deposition technique. Ni–Co alloy and nanocomposite coatings deposited from the SCD technique have exhibited superior properties of higher Co content and higher nanoparticle content which translate to better microhardness, and improved wear and corrosion resistance of the deposited coatings. As such, selection of SCD in DC electrodeposition of Ni–Co composites should be considered for further research. Based on current trends, it can be seen that owing to their exceptional wear resistance and corrosion properties, deposited Ni–Co alloys and their nanocomposites are strong contenders for further application in the aviation industry, for use in jet engine fabrication, automotive engineering, textiles and general engineering. In recent years, a keen interest has developed in specialized engineering where deposited coatings consist of mixed functional properties, as well as deposition of superhydrophobic surface coatings which exhibit excellent wear and corrosion resistance, better self-cleaning and good tribological properties.

In essence, nanoparticles can be selected to match the desired properties of any coating, and with such capacity for discovery coupled with the ever-growing need of better material properties, the possibilities for future applications are endless.
